# Differentiating Tuberculous and Pyogenic Spondylodiscitis: Part I—Epidemiology, Clinical Features, Laboratory Markers, and Tissue-Based Diagnosis

**DOI:** 10.3390/diagnostics16142243

**Published:** 2026-07-17

**Authors:** Anamaria Marian, Oana Maria Vanța, Valentin Danci, Larisa Rotaru, Maria-Magdalena Tămaș, Rodica Ungur, Simona Rednic, Cristina Pamfil

**Affiliations:** 1 Department of Rheumatology, “Iuliu Hatieganu” University of Medicine and Pharmacy, 400012 Cluj-Napoca, Romania; anymaria.marian@gmail.com (A.M.); mm_tamas@yahoo.com (M.-M.T.); srednic.umfcluj@gmail.com (S.R.); cristinapamfil.umfcluj@gmail.com (C.P.); 2 Emergency Clinical County Hospital, 400012 Cluj-Napoca, Romania; 3 Department of Neurology, “Iuliu Hatieganu” University of Medicine and Pharmacy, 400012 Cluj-Napoca, Romania; 4 Department of Medical Genetics, “Iuliu Hatieganu” University of Medicine and Pharmacy, 400012 Cluj-Napoca, Romania; valentin.adri.danci@elearn.umfcluj.ro; 5 Nicolae Testemiţanu Internal Medicine Department, State University of Medicine and Pharmacy, 2004 Chișinău, Moldova; larisa.rotaru@usmf.md; 6 Department of Medical Specialties, Faculty of Medicine, “Iuliu Hațieganu” University of Medicine and Pharmacy, 400012 Cluj-Napoca, Romania; ungurmed@yahoo.com

**Keywords:** tuberculous spondylodiscitis, pyogenic spondylodiscitis, spinal tuberculosis, vertebral osteomyelitis, biopsy, histopathology, Xpert MTB/RIF Ultra, molecular diagnosis, culture-negative infection, differential diagnosis

## Abstract

Distinguishing tuberculous spondylodiscitis (TS) from pyogenic spondylodiscitis (PS) remains difficult when presentation is non-specific, blood cultures are negative, or initial biopsy is non-diagnostic. The two entities differ substantially in antimicrobial strategies, resistance testing requirements, public health interventions, and surgical thresholds, yet diagnostic delay is associated with neurological deficits, spinal instability, and permanent deformity. This narrative review maps the non-imaging evidence most useful for frontline differentiation between TS and PS across five domains: epidemiology and risk stratification, clinical presentation, laboratory markers, tissue acquisition and histopathology, and molecular diagnostics. PubMed/MEDLINE was searched from inception to 31 March 2026 using pre-specified Boolean search terms; a secondary Scopus search identified no additional eligible records. Following screening, approximately 90 records were included in this synthesis. Priority was given to comparative TS-versus-PS cohorts, biopsy-yield and culture-negative studies, pathology series, pediatric data, and recent molecular diagnostics literature. Epidemiological TB (tuberculosis) risk, longer symptom duration, constitutional symptoms, deformity, and a less intense acute-phase response increase the probability of TS, whereas healthcare exposure, bacteraemia, recent spinal procedures, and brisk neutrophilic inflammation favor PS. In stable patients, the highest-yield strategy is early blood cultures followed by image-guided biopsy with parallel tissue allocation for bacterial culture, mycobacterial studies, histopathology, and selected molecular assays. No single laboratory marker reliably distinguishes TS from PS without tissue confirmation. Per a 2023 systematic review and meta-analysis, image-guided percutaneous biopsy achieves microbiological confirmation in approximately one-third of cases. Histopathology demonstrating caseating granulomatous inflammation supports TS, although a substantial minority of confirmed cases lack classic features. Supported by cohort prospective data, Xpert MTB/RIF Ultra has the clearest first-line molecular role when TS is plausible and should be requested at the time of first biopsy rather than reserved for salvage testing; broader or targeted next-generation sequencing is best reserved for selected unresolved cases. Imaging differentiation is addressed in the companion manuscript, Part II.

## 1. Introduction

Infectious spondylodiscitis is an increasingly recognized cause of destructive back pain in adults and children, and its incidence has risen in contemporary series as populations age, comorbidity burdens increase, and spinal imaging becomes more accessible [1,2,3,4,5,6,7]. The two major etiological categories relevant to routine clinical practice are pyogenic spondylodiscitis (PS) and tuberculous spondylodiscitis (TS) [1,2,8,9]. Tuberculosis remains the commonest cause of granulomatous spinal infection, and the spine is the predominant site of musculoskeletal tuberculosis [2,10,11,12,13,14,15,16,17].

Initial presentation is frequently non-specific, typically comprising persistent spinal pain, variably elevated inflammatory markers, and imaging findings compatible with infection but not pathognomonic for a single organism [2,3,8]. Distinguishing them early can be challenging and is not simply taxonomic. The two entities differ substantially in antimicrobial strategy and duration, requirements for resistance testing and public health intervention, and thresholds for surgical management. Diagnostic delay is associated with increased risk of neurological deficit, spinal instability, and permanent deformity [2,7,18,19]. Diagnostic uncertainty is particularly pronounced when blood cultures are negative, when prior empiric antibiotics reduce microbiological yield, or when biopsy is delayed or non-diagnostic [2,7,20]. Even with computed tomography (CT)-guided techniques, pathogen isolation is achieved in only a minority of cases [21,22,23]. Without microbiological confirmation, accurate diagnosis relies on integrating clinical, laboratory, and imaging findings [2,18,19,20,24].

Several narrative and systematic reviews have addressed spondylodiscitis diagnosis, but none has integrated all five non-imaging diagnostic domains—epidemiology and pre-test probability, clinical features, laboratory markers, tissue acquisition and histopathology, and molecular diagnostics—within a single clinically structured framework oriented specifically toward culture-negative or biopsy-delayed disease, which represents the most diagnostically challenging subset encountered in routine practice. Existing reviews either address imaging and non-imaging evidence jointly without adequate depth in either domain or focus on a single modality or pathogen type. The present review addresses this gap by synthesizing evidence across all five non-imaging domains around the clinical decision points most relevant to frontline practitioners.

This manuscript constitutes Part I of a two-part paired review. The non-imaging and imaging evidence bases for TS-versus-PS differentiation are each sufficiently large, methodologically distinct, and clinically nuanced to warrant independent synthesis; combining them in a single manuscript would require either sacrificing depth or producing a document of an unsuitable length for a journal article. The two parts are designed to be read together as complementary components of an integrated diagnostic framework: Part I provides the pre-imaging probabilistic foundation, and Part II maps the imaging phenotype that modifies that probability and guides biopsy targeting. Readers applying either part in isolation should be aware that TS-versus-PS differentiation in clinical practice requires integration of both imaging and non-imaging evidence within a Bayesian framework.

## 2. Materials and Methods

This manuscript is Part I of a two-part narrative review examining the differential diagnosis of tuberculous spondylodiscitis (TS) and pyogenic spondylodiscitis (PS) in adults and children. The narrative format was chosen because the available literature is heterogeneous in study design, case definitions, reference standards, and endemic settings, precluding meaningful statistical pooling of most diagnostic parameters of interest.

Searches of PubMed/MEDLINE were conducted from database inception to 31 March 2026 using combinations of the following terms across three concept blocks: (1) condition—spondylodiscitis, vertebral osteomyelitis, spinal tuberculosis, pyogenic spondylodiscitis, discitis; (2) pathogen or aetiology—Mycobacterium tuberculosis, pyogenic, granulomatous, Staphylococcus, Kingella; and (3) diagnostic domain—biopsy, histopathology, culture, Xpert Ultra, 16S polymerase chain reaction, ribosomal RNA gene (16S rRNA PCR, metagenomic next-generation sequencing (mNGS), interferon-gamma release assay (IGRA), C-reactive protein (CRP), erythrocyte sedimentation rate (ESR). A dedicated sub-search appended pediatric age-group terms to the combined string. A secondary search of Scopus was performed using identical terms and date range; no additional eligible records were identified beyond those retrieved through PubMed/MEDLINE. Reference lists of included comparative studies, biopsy-yield cohorts, and five high-citation narrative reviews published after 2015 were systematically hand-searched [9,25,26,27,28] by two co-authors to identify additional relevant records; these sources are listed in Supplementary File S1, which also provides the full reproducible Boolean search strings. No protocol was prospectively registered, consistent with the narrative review design; the review question and eligibility criteria were defined prior to searching. Title and abstract screening and full-text eligibility assessment were performed by the lead authors (O.M.V. and A.M.). Eligibility decisions were reviewed for consistency on a 20% random sample of screened records by a co-author (C.P.), with discordant decisions resolved by discussion. Formal dual independent screening of all records was not performed, which is acknowledged as a limitation of the narrative review design.

Because this article was designed as a narrative review rather than a systematic review, the literature search was conducted iteratively and by topic to support the manuscript’s predefined diagnostic domains. Formal de-duplicated title and abstract screening of all records retrieved by broad database searches was not performed; accordingly, the article is not presented as compliant with the Preferred Reporting Items for Systematic Reviews and Meta-Analyses (PRISMA) guidelines. To improve transparency and reproducibility, the Methods section has been expanded, and the full electronic search strategies are provided in Supplementary File S1.

Records were considered eligible if they reported clinical, laboratory, histopathological, or molecular diagnostic data in patients with confirmed or probable spondylodiscitis, or if they directly compared TS and PS on at least one diagnostic parameter. For the purposes of eligibility assessment, confirmed spondylodiscitis was defined as a microbiologically established disease by a positive culture or a validated molecular test from spinal or paraspinal tissue or blood; probable spondylodiscitis was defined as a clinical and radiological diagnosis meeting the Infectious Diseases Society of America (IDSA) 2015 or Société de Pathologie Infectieuse de Langue Française (SPILF) 2022 guideline criteria in the absence of microbiological confirmation. Case reports and series of fewer than ten patients were excluded unless they reported findings not available in larger studies. Imaging-only studies are addressed in Part II and were not included here.

Evidence selection was purposive, with studies prioritised according to the following hierarchy: (1) comparative TS-versus-PS cohort studies with microbiological reference standards; (2) systematic reviews and meta-analyses; (3) prospective or consecutively enrolled cohort studies; (4) biopsy-yield and culture-negative infection studies; (5) pathology and histopathology series; (6) pediatric series; and (7) molecular diagnostics literature including recent next-generation sequencing studies. The pathobiological basis of imaging and phenotypic differences between TS and PS is addressed as contextual background within the epidemiology and risk stratification domain rather than as a separate evidence domain. Formal risk-of-bias scoring was not performed. Instead, studies are characterized throughout the Results according to three dimensions that affect the credibility and generalisability of their findings: study design (prospective vs. retrospective; consecutive vs. selected enrolment), reference standard (microbiologically confirmed vs. clinically probable diagnosis), and population representativeness (single-center tertiary vs. multicentre or population-based data). This qualification is applied where it materially affects the interpretation of reported estimates; single-center retrospective cohorts from tertiary referral centers are noted as potentially subject to referral bias and may not reflect the diagnostic performance achievable in district-level or primary care practice. Quantitative estimates of diagnostic performance are presented as reported in primary studies and are not pooled; where multiple studies report discordant results for the same parameter, the range of estimates is stated alongside an explicit explanation of likely sources of discordance. Evidence strength is characterized throughout using three informal conventions: *consistent evidence* indicates findings replicated across at least three independent cohorts with compatible designs; *limited evidence* indicates findings from one or two studies or from studies with important methodological limitations; and *insufficient evidence* indicates that no eligible data were identified or that available data were too heterogeneous to support a directional conclusion. Sub-threshold studies—case reports or series of fewer than ten patients—are cited only where they document diagnostic findings not available in larger series and are identified as such in the text. The review is weighted towards adult practice; pediatric studies are used to characterize age-specific epidemiological and clinical patterns rather than to construct a separate diagnostic algorithm.

The final reference set included 90 records selected for their relevance to the diagnostic differentiation between TS and PS. To provide a numerical overview of the literature base, records were grouped according to their primary role in the narrative synthesis: reviews, guidelines, and contextual diagnostic or management sources (*n* = 20); epidemiology and clinical cohort studies (*n* = 18); comparative TS-versus-PS or pathogen-comparative studies and diagnostic models (*n* = 13); biopsy-yield, culture-yield, and culture-negative vertebral infection studies or reviews (*n* = 8); histopathology, pathology, and acid-fast staining sources (*n* = 5); pediatric spinal infection studies or reviews (*n* = 5); laboratory, microbiological, molecular, and immunological diagnostic method studies or reviews (*n* = 18); and imaging-focused articles cited only for contextual linkage with the companion imaging review (*n* = 3). Categories were assigned based on each reference’s primary role in the manuscript, recognizing that some records overlap thematically.

## 3. Results

### 3.1. Epidemiology and Risk Stratification

The background epidemiology of vertebral infection is important because it frames the pre-test probability before any tissue result is available. Across population-based studies from Europe and Japan, native vertebral osteomyelitis has increased steadily over the last 15 years, particularly in older adults with diabetes, malignancy, renal disease, immunosuppression, or prior spinal intervention [4,5,6,29,30]. In this overall population, PS remains the dominant cause. By contrast, TS is concentrated in patients with clear epidemiological tuberculosis (TB) risk: birth in or recent residence in a high-incidence country, previous tuberculosis, known TB contact, human immunodeficiency virus (HIV) infection, severe immunosuppression, or radiological or microbiological evidence of concomitant pulmonary or extra-spinal TB [12,13,14,15,16,31].

Spondylodiscitis, whether pyogenic or tuberculous, primarily affects adults, with most cases occurring in individuals over 50 years of age [3,4,5,18,30,32,33]. Several comparative cohorts suggest that patients with TS are, on average, younger than those with PS, especially in migrant or HIV-associated populations; however, the overlap is substantial, and younger age should be interpreted only as a shift in probability rather than as a discriminator [18,19,24,31,34,35]. In low-incidence countries, clinicians should think of TS both in younger adults from TB-endemic settings and in older native adults with reactivation risk factors. Conversely, older age, healthcare exposure, recent bacteremia, infective endocarditis, urinary sepsis, skin and soft-tissue infection, or a recent spinal procedure all strengthen the prior probability of PS [4,5,6,7,35,36,37]. Risk factors should be interpreted within a structured probabilistic framework rather than as isolated discriminators, as they represent distinct domains of host susceptibility, exposure history, and associated clinical conditions. A structured synthesis is presented in Table 1. The evidential weight is uneven: TB exposure or endemic origin, recent spinal procedures, prior bacteremia, and infective endocarditis are supported more consistently across comparative cohorts and carry meaningful discriminatory weight in pre-test probability estimation. By contrast, anticoagulant use and vitamin D deficiency have been reported as associated with spondylodiscitis in some series, but consistent evidence supporting their value as discriminators between TS and PS is absent. These factors are therefore classified as insufficient evidence for diagnostic discrimination and are retained in Table 1 solely to contextualize their appearance in the comparative literature and to prevent over-interpretation; they should not be used as diagnostic or probabilistic discriminators in clinical practice.

Sex distribution in spondylodiscitis shows a slight male predominance, regardless of etiology, but has minimal value in differentiating tuberculous (TS) from pyogenic spondylodiscitis (PS) [4,24,32,33]. Most comparative studies demonstrate no significant sex-based differences [20,34,38,39,40]. However, Akiyama et al. reported a modest but significantly higher male prevalence in PS compared with TS (59.3% vs. 50.2%, *p* = 0.001), a finding supported by Kim et al. and Yoon et al. [5,18,19].

#### Spondylodiscitis in Children

Pediatric spondylodiscitis follows a distinct epidemiological pattern. Pediatric spondylodiscitis is rare, accounting for only a small fraction (1–2%) of childhood osteomyelitis, and its age distribution is bimodal, with one group in early childhood and another in later childhood or adolescence [41,42,43,44,45]. Tuberculous cases are more likely in children from endemic settings, with household TB exposure, malnutrition, overcrowding, incomplete vaccination coverage, or immune compromise [43,44,45]. In the retrospective pediatric series by Roversi et al., tuberculous cases were older and were associated with more sequelae than non-tuberculous cases [43]. By contrast, pyogenic pediatric disease more often arises from transient bacteremia, and microbiology in published pediatric reviews is dominated by *Staphylococcus aureus* and *Kingella kingae* rather than *Mycobacterium tuberculosis* [42,43,44].

**Table 1:** Epidemiological and clinical factors influencing the differential diagnosis between pyogenic and tuberculous spondylodiscitis.

Domain	Factor	Association	Interpretation for Differential Diagnosis	Clinical Implication
**Host factors**	Diabetes mellitus	Favors PS	Increased susceptibility to pyogenic bacteremia	Prioritize blood cultures and infection source search
	Hemodialysis	Favors PS	Repeated vascular access, healthcare exposure	Consider nosocomial pathogens
	Cardiovascular disease	Favors PS	Associated with endovascular infection	Evaluate for infective endocarditis
	HIV infection	Depends on CD4 count	Intermediate immunosuppression favors TS; advanced immunosuppression broadens the spectrum	Tailor microbiological work-up (TB, atypical, fungal)
	Malignancy	No clear difference	Predisposes to multiple infection types	Broaden microbiological work-up
	Rheumatic disease	No clear difference	Often reflects immunosuppression	Consider opportunistic pathogens
	Chronic kidney disease	No clear difference	Limited discriminatory value	Interpret in conjunction with other risk factors
**Exposure-related factors**	Prior spinal surgery	Strongly favors PS	Direct inoculation or postoperative infection	Early imaging and bacteriological evaluation
	Epidural procedures	Favors PS	Procedure-related infection	Consider healthcare-associated etiology
	Intravenous drug use	Favors PS	Hematogenous spread	Evaluate for *S. aureus* and endocarditis
	Prior bacteraemia	Strongly favors PS	Indicates hematogenous seeding	Blood cultures may be diagnostic
	TB exposure/endemic origin	Strongly favors TS	Key epidemiological determinant	Early biopsy with TB PCR/culture
**Associated conditions**	Infective endocarditis	Strongly favors PS	Established bacteremia source	Mandatory cardiac evaluation
	Anticoagulant use	Weak association with PS	Likely surrogate for comorbidity	Low-weight contextual factor
**Nutritional factors**	Vitamin D deficiency	Weak association with TS	Possible impaired antimycobacterial response	Supportive but non-specific, low-weight factor

Data synthesized from comparative cohort and observational studies [4,5,18,19,20,24,30,31,32,46,47,48,49,50,51,52,53]. Evidential weight across risk factors is uneven. TB exposure or endemic origin, prior bacteremia, infective endocarditis, and recent spinal procedures are supported more consistently across independent cohorts and carry meaningful discriminatory weight in pre-test probability estimation. Anticoagulant use and vitamin D deficiency have been reported in some series, but consistent evidence supporting their value as discriminators between TS and PS is absent; these factors are classified as insufficient evidence for diagnostic discrimination and are included in the table solely to contextualize their appearance in the comparative literature. They should not be used as diagnostic or probabilistic discriminators in clinical practice. CD4 (cluster of differentiation 4); HIV (human immunodeficiency virus); PCR (polymerase chain reaction); PS (pyogenic spondylodiscitis); TS (tuberculous spondylodiscitis); TB (tuberculosis); *S. aureus* (*Staphylococcus aureus*).

### 3.2. Pathobiology Relevant to Phenotype

The differing phenotype of TS and PS reflects both vascular anatomy and pathogen biology. In adults, spinal infection is usually hematogenous, with seeding of the subchondral endplate; the paravertebral venous plexus, as described by Batson, may contribute to the thoracic predilection, multilevel spread, and occasional noncontiguous lesions seen in spinal tuberculosis [17,37,54,55,56]. In children, retained disc vascularity helps to explain why discitis may present as a primary process rather than exclusively as secondary endplate extension [43,44,45].

At the bedside, the clinically relevant distinction is that Mycobacterium tuberculosis usually produces a paucibacillary granulomatous infection with slow progression, caseous necrosis, subligamentous spread, and large cold abscesses, whereas pyogenic organisms—most commonly Staphylococcus aureus—provoke acute neutrophilic inflammation, earlier disc and endplate destruction, and a more marked systemic inflammatory response [2,11,17,37,54,57,58]. The practical consequence is a longer prodrome, greater deformity burden and less striking inflammatory biology in TS, compared with the more acute inflammatory phenotype usually seen in PS.

### 3.3. Clinical Presentation

Back pain is the dominant symptom in both entities, occurring in 67–100% of cases, and is insufficient for differentiation on its own [1,3,58]. Pain may be mechanical or inflammatory in character and can be associated with radicular symptoms, but comparative studies have not identified consistent differences in pain pattern between TS and PS [1,3,18,24,31,32,46]. Spinal tenderness, present in up to 78–97% of patients, is the most frequent clinical sign and is often accompanied by paraspinal spasm and restricted mobility [1,3,55]. What usually separates TS from PS is disease tempo. Across comparative adult cohorts, TS shows a substantially longer symptomatic phase before diagnosis than PS, often measured in weeks to months rather than days to a few weeks [18,19,24,35,36,46,59]. This prolonged course should not reassure clinicians. Delayed presentation is one reason TS is over-represented among patients with vertebral collapse, angular deformity, large paraspinal collections, and fixed neurological deficits [18,19,24,35,36,48].

Systemic features are helpful but imperfect discriminators. Fever occurs in approximately half of cases and is less frequent in TS than in PS, particularly in non-disseminated disease [1,13,18,24,53]. When present in TS, fever often reflects concomitant extraspinal or disseminated tuberculosis [12]. Constitutional symptoms such as weight loss, night sweats, anorexia, or evidence of pulmonary tuberculosis increase the likelihood of TS [3,12,13,18,31,60,61]. The overlap remains clinically important: culture-negative PS may also present with less fever and lower inflammatory markers than culture-positive PS, so an apparently indolent presentation should not be taken as evidence of tuberculosis [20,62].

Neurological involvement deserves particular emphasis because it often drives urgency and prognosis. Radiculopathy, weakness, cord compression, and sphincter symptoms occur in both TS and PS, but comparative studies generally report a higher frequency of neurological deficit and visible deformity in TS, probably because of delayed diagnosis and more extensive bony destruction by the time of presentation [18,19,24,38,46]. Extraspinal clues can be useful: psoas abscess, multifocal TB, chronic cough, or lymphadenopathy all increase the likelihood that spinal disease is tuberculous rather than pyogenic [12,13,14,15,16,18].

#### Clinical Features in Children

In children, presentation is even less specific. Irritability, refusal to sit or walk, limp, abdominal pain, or reduced spinal movement may predominate, while focal back pain and fever may be absent [42,43,45]. The insidious phenotype of pediatric TS is particularly hazardous because delayed recognition in the growing spine may lead rapidly to vertebral collapse, progressive kyphosis, and neurological compromise [43].

Although detailed imaging differentiation is addressed in Part II of this review, clinicians should be aware that two imaging features in particular—multilevel non-contiguous vertebral involvement and large thin-walled paravertebral collections—may be visible on initial MRI and, when present, substantially increase the pre-test probability of TS before tissue results are available [reference to Part II]. These findings should prompt consideration of whole-spine staging and early mycobacterial tissue processing even when the clinical and laboratory picture is equivocal.

### 3.4. Laboratory Markers

Routine inflammatory markers are helpful in recognizing spinal infection, but their discriminatory value remains probabilistic rather than definitive. ESR and CRP are usually abnormal in both TS and PS, whereas the total leukocyte count is less sensitive overall [1,35,37,56,58]. Consistent evidence across multiple comparative cohorts suggests that PS is associated with a more pronounced inflammatory response [2,24,31,39,46]. In the Korean comparative cohort by Kim et al., leukocytosis, CRP elevation, and ESR > 40 mm/h were all significantly more common in PS than in TS [19]. More recently, the 2026 propensity score-matched analysis by Bulut et al. showed that white blood cell (WBC) count, neutrophil count, ESR, and CRP were all higher in PS, even after adjustment for age, sex, and comorbidity burden [63]. Beyond etiology, the magnitude of the inflammatory response is also influenced by microbiological status: microbiologically confirmed infections more often show marked elevations in WBC count and CRP, whereas culture-negative cases tend to have a more attenuated profile [32,62,64]. This distinction matters clinically because culture-negative PS may overlap with the less inflammatory phenotype of TS.

Newer composite hematological markers are attractive because they are inexpensive and immediately available. The best studied is the neutrophil-to-lymphocyte ratio (NLR). Limited evidence from a single-center retrospective study suggests that NLR may offer incremental discriminatory value: in the 2022 cohort by Liu et al., NLR was markedly lower in spinal tuberculosis than in pyogenic spinal infection, and a threshold below 6.742 achieved a sensitivity of 78.3% and specificity of 83.6% for TS [40]. Insufficient evidence currently supports the platelet-to-lymphocyte ratio (PLR) as a clinically useful discriminator, but showing a lower diagnostic performance [40]. Additional models have incorporated serum albumin and fibrinogen, with lower albumin and higher fibrinogen tending to favor PS rather than TS [18,38]. Several important caveats apply to the clinical use of NLR and PLR. Both markers derive from retrospective single-center cohorts without external validation in independent populations, and the reported threshold values (NLR < 6.742 for TS in the Liu et al. [40] are likely to be sensitive to local case mix, TB prevalence, and the proportion of immunocompromised patients in the study population. The overestimation risk inherent in single-center derivation studies is well recognized in diagnostic biomarker literature, and external validation in geographically and epidemiologically diverse cohorts is required before either marker can be recommended as a routine clinical discriminator. At present, NLR and PLR should be regarded as hypothesis-generating findings that may add incremental value when interpreted alongside conventional markers (CRP, ESR, WBC) rather than as stand-alone discriminators.

Limited evidence, primarily from single-center series, suggests that procalcitonin is best interpreted as a marker that increases confidence in pyogenic infection when elevated, rather than as a test that excludes tuberculosis when low. Median concentrations are usually higher in PS than in TS, but the overlap is considerable and discriminatory performance is modest [18,65]. Thus, no single marker—CRP, ESR, WBC, NLR, albumin, fibrinogen, or procalcitonin—should be used to decide whether prolonged anti-tuberculous therapy or anti-staphylococcal therapy is warranted without tissue confirmation.

Limited evidence supports the role of Interferon-gamma release assays (IGRAs) as an adjunctive test. A positive IGRA can support the prior probability of TS when clinical and radiological suspicion is already high, particularly in low-incidence settings, but it cannot distinguish latent from active TB and loses specificity in populations with a high background prevalence of latent infection [20,66]. In a recent prospective study, QuantiFERON-TB Gold showed moderate diagnostic accuracy in spinal tuberculosis (sensitivity 82.6%, specificity 77.8%). Although performance improved when combined with mucosal-associated invariant T-cell phenotyping, this approach remains investigational and is not part of routine practice [66]. Clinically, IGRAs should therefore be regarded as adjunctive immunological clues rather than diagnostic endpoints. Table 2 summarizes the main clinical and biological clues favoring tuberculous versus pyogenic spondylodiscitis.

### 3.5. Tissue Acquisition and Cultures

Tissue acquisition and microbiological identification remain central to differentiation, as clinical and laboratory findings overlap substantially. The biopsy-first pathway described here applies to hemodynamically stable patients without sepsis, rapidly progressive neurological deterioration, or another indication for immediate decompression or stabilization. In urgent settings, antimicrobial therapy and surgery may need to proceed in parallel with attempts to secure microbiological material [7,25]. In stable patients, empiric antimicrobial therapy is generally deferred until blood cultures and biopsy are obtained, whenever feasible, to optimize microbiological yield [7,25].

A comprehensive microbiological work-up should include blood cultures, evaluation of potential primary infection sources, and, when feasible, tissue sampling from vertebral or paraspinal sites [7,52]. Blood cultures should be obtained in all patients prior to antimicrobial therapy, as they may establish a pyogenic etiology without the need for spinal sampling, particularly in the presence of Staphylococcus aureus or Gram-negative bacteremia [7,9]. However, blood cultures are much less helpful for TS, in which microbiological confirmation usually depends on biopsy of spinal or paraspinal tissue. Conventional mycobacterial culture remains important for definitive identification and phenotypic susceptibility testing, but it is slow and comparatively insensitive in paucibacillary bone disease [11,17,67].

Across studies, tissue cultures generally demonstrate higher sensitivity than blood cultures, although results are heterogeneous, and no single sampling strategy guarantees pathogen isolation [21,47]. Histopathology further increases diagnostic yield and may be more informative than culture alone, particularly in culture-negative cases, underscoring that biopsy should be approached as both a microbiological and pathological procedure [68].

Image-guided percutaneous biopsy—most commonly CT-guided, but also fluoroscopy- or magnetic resonance imaging (MRI)-guided—is the preferred initial invasive approach, as it is less morbid than open biopsy and allows targeted sampling of the disc, endplate, paraspinal soft tissue, or drainable collections [7,9,69,70]. However, clinicians should not overestimate its culture yield. A 2023 systematic review and meta-analysis demonstrated that image-guided biopsy yields microbiological confirmation in approximately one-third of cases, with considerable heterogeneity related to biopsy target, prior antimicrobial exposure, and reference standards [70]. Earlier cohort studies and systematic reviews have demonstrated similarly heterogeneous diagnostic yields, reinforcing that a negative percutaneous biopsy does not exclude infection and cannot reliably exclude tuberculosis [21,69].

Procedural and clinical factors further influence diagnostic performance. Prior antimicrobial therapy is associated with reduced microbiological yield, whereas successful fluid aspiration increases the likelihood of pathogen identification; an antibiotic-free interval of several days (approximately ≥4 days) may improve yield when clinically feasible [71].

When the first biopsy is non-diagnostic, subsequent steps depend on clinical stability and the strength of residual suspicion. In stable patients with persistent radiological concern, repeat percutaneous biopsy or open biopsy is preferable to prolonged empiric treatment, particularly when TS or another atypical infection remains plausible [7,64,69]. Open biopsy is associated with higher diagnostic yield in many series and is particularly appropriate when surgical decompression or stabilization is already indicated [7]. Specimen handling should be deliberate from the outset, with parallel requests for routine bacterial and mycobacterial culture, histopathology, and, where specifically indicated by the clinical context, additional fungal or molecular studies. This integrated approach improves diagnostic yield in culture-negative cases and reduces loss of diagnostic material through sequential testing [7,64,68].

### 3.6. Histopathology

Histopathology is particularly valuable in culture-negative or partially treated disease because it complements microbiology by defining the pattern of tissue injury. In this context, histology is especially useful for differentiating pyogenic from granulomatous infection and for determining whether the biopsy material is truly compatible with infection rather than contamination or non-infectious mimics [1,9,72].

In spinal tuberculosis, the classical pattern is granulomatous inflammation with epithelioid histiocytes, multinucleated giant cells, tubercle formation, and caseous necrosis [17,72,73]. In the pathological series by Li et al., which analyzed 181 specimens, typical TB-associated features were present in 80.7% of cases, with caseous necrosis, multinucleated giant cells, granulomatous inflammation, and tubercles being the dominant findings [72]. Sequestrum with relatively limited reparative new bone formation may also support TS over PS [72]. Conversely, PS is typically characterized by dense neutrophilic inflammation, purulent necrosis without caseation, and destructive osteomyelitis without a granulomatous response [9,74,75].

In clinical practice, this distinction is helpful but not absolute. Two cautions are particularly relevant. First, histological tuberculosis is not synonymous with caseation and granulomatous inflammation: a substantial minority of proven TS cases lack classic caseating granulomas, while granuloma formation may also occur in selected mimics outside the core TS-versus-PS differential, including fungal infection and brucellosis [10,12,72,73]. Second, rare atypical or suppurative forms of TS may histologically resemble pyogenic infection, with little or no granuloma formation, particularly in small biopsies or partially treated disease [72,76]. In such cases, histology alone may not resolve the differential. Direct acid-fast staining can support TS when positive, but its sensitivity in spinal tissue is limited by low bacillary burden [10,11]. Even with improved fluorescent techniques, a negative stain cannot exclude TS [77]. The practical point is that histology should be interpreted alongside stain, culture, and molecular data rather than in isolation.

### 3.7. Molecular Diagnosis

**Figure 1:**
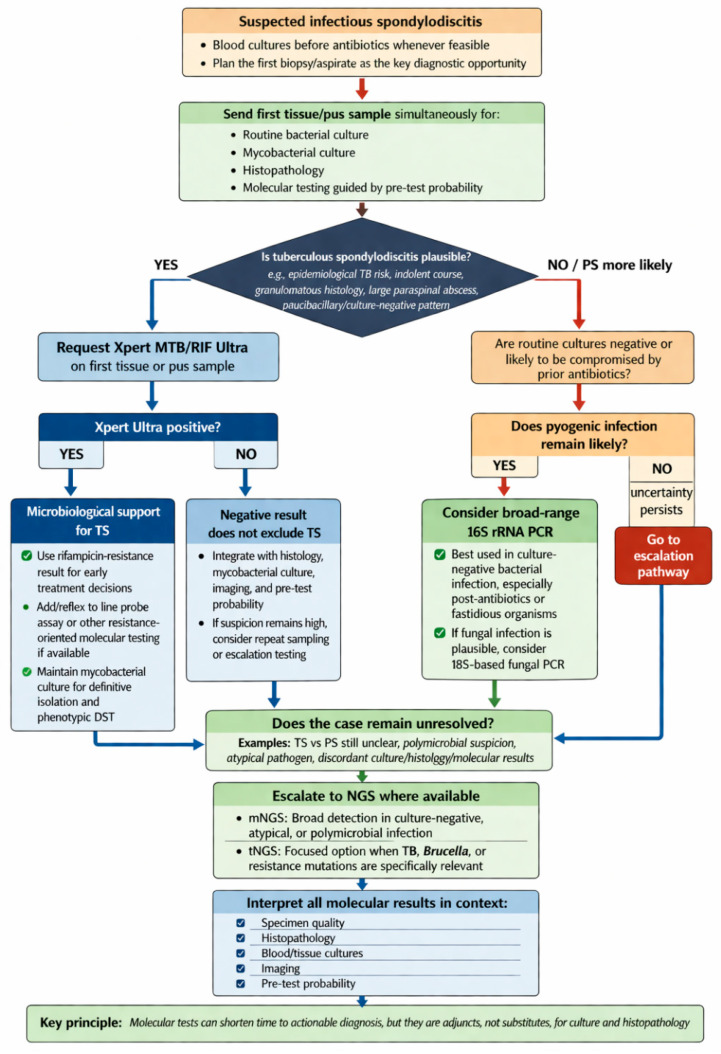
Proposed molecular diagnostic pathway for clinically stable patients with suspected spondylodiscitis. This figure presents an evidence-informed expert synthesis integrating microbiological, histopathological, and molecular approaches in suspected infectious spondylodiscitis. The pathway is centered on the TS-versus-PS differential in clinically stable patients and outlines selective escalation strategies for unresolved cases; selected atypical targets shown within the escalation boxes are illustrative rather than exhaustive. This algorithm has not been prospectively validated and should be interpreted as a structured clinical decision aid reflecting current evidence and expert synthesis, rather than as an evidence-based clinical guideline Molecular results should always be interpreted in the context of clinical, radiological, and conventional microbiological findings The pathway should be adapted to local resource availability, as metagenomic and targeted next-generation sequencing approaches are not available in all settings [7,9,25,78,79,80,81,82]. PS (pyogenic spondylodiscitis); TS (tuberculous spondylodiscitis); TB (tuberculosis); 16S rRNA PCR (16S ribosomal RNA gene polymerase chain reaction); NGS (next-generation sequencing); mNGS (metagenomic next-generation sequencing); tNGS (targeted next-generation sequencing).

Molecular testing is most useful when conventional methods are either too slow or too insensitive to answer the immediate clinical question. In practice, this is chiefly the case when TS is plausible and rapid confirmation with an early rifampicin-resistance signal would alter management, or when PS remains likely despite negative or compromised cultures, particularly after prior antibiotic exposure. The evidence base is not equivalent across assays. Support is strongest for rapid TB nucleic acid amplification on first-line tissue when TS is suspected; evidence for broader sequencing platforms or extended resistance assays in spinal tissue is more limited and should be interpreted more cautiously. Molecular tests should therefore be used as adjuncts within a structured diagnostic pathway rather than as replacements for culture or histopathology [9,64,78] and should always be interpreted in the context of clinical, radiological, and conventional microbiological findings [9,25,78,79,80,81,82]. The detailed utility and limitations of molecular tests are summarized in Table 3, and a pragmatic stepwise approach to their use is outlined in Figure 1.

For suspected TS, the molecular test with the clearest immediate clinical value is a rapid pathogen-specific nucleic acid amplification assay, particularly Xpert MTB/RIF Ultra, where available [11,78,83,84]. This reflects the practical challenges of spinal tuberculosis, which is usually paucibacillary, difficult to sample repeatedly, and often associated with delayed microbiological confirmation and delayed information on resistance [10,11,17]. Current World Health Organization (WHO) guidance supports the use of Xpert MTB/RIF or Xpert Ultra for extrapulmonary TB and rifampicin-resistance detection, with follow-on molecular resistance testing considered once Mycobacterium tuberculosis has been identified [78]. In spinal disease, the practical implication is that the initial tissue sample should be used efficiently, with simultaneous requests for mycobacterial culture, histopathology, and rapid molecular testing rather than a sequential salvage approach [7,25,70].

Consistent with WHO guidance [78], a prospective single-center study by Waters et al. evaluated Xpert MTB/RIF Ultra in spinal tissue and reported high diagnostic accuracy, with sensitivities of 100% (95% confidence interval [CI], 69–100%) in open biopsy specimens and 89% (95% CI, 65–99%) in CT-guided samples, outperforming culture, particularly in CT-guided biopsies [67]. These findings support the use of Xpert Ultra at the time of the first biopsy rather than as a salvage test; however, the single-center design and modest sample size mean that the estimates should be interpreted as indicative of the order of magnitude of diagnostic gain rather than as precise, calibrated values. Similar results have been reported in osteoarticular TB, where Xpert MTB/RIF Ultra was more sensitive than the original Xpert MTB/RIF assay while maintaining high specificity [85]. When TS is a realistic possibility, Xpert MTB/RIF Ultra should therefore usually be requested at the time of first biopsy or aspirate rather than reserved for salvage testing. A negative result, however, does not reliably exclude TS in paucibacillary disease or limited sampling [67,78].

Once tuberculosis has been established, the next question is whether early information on resistance will alter management. Line probe assays can extend resistance detection beyond rifampicin and have well-established accuracy in pulmonary tuberculosis, particularly in smear-positive disease; however, extrapulmonary and spinal data remain limited and depend on bacillary burden and specimen quality [11,78,86]. In spinal practice, they are best regarded as selective follow-on tests after TB detection—particularly when multidrug resistance is suspected or early regimen optimization is needed—rather than as routine first-line assays for spinal tissue.

In suspected PS, the most relevant molecular adjunct is broad-range bacterial PCR, usually targeting the 16S rRNA gene and coupled with sequencing when required. Its principal role is in culture-negative infection, especially after prior antibiotic exposure or when fastidious organisms are suspected [8,79,87]. Because it detects bacterial deoxyribonucleic acid (DNA) rather than viable organisms, 16S rRNA PCR may remain positive despite negative conventional cultures. Observational studies suggest an incremental diagnostic yield when 16S rRNA PCR is added to culture, particularly after prior antibiotic use [79,87,88]. Its limitations are equally important: contamination may generate false-positive results, clinically irrelevant DNA may be overinterpreted, and susceptibility information is not provided [9,79].

Beyond targeted assays, next-generation sequencing is best regarded as a selective escalation step rather than routine first-line testing. Metagenomic next-generation sequencing (mNGS) offers broad, hypothesis-free detection and may be helpful in culture-negative, polymicrobial, or diagnostically unresolved infection [80,89,90,91]. However, interpretation is constrained by contamination, host DNA background, incomplete susceptibility inference, cost, and variable laboratory availability [80,91]. Although recent pooled estimates suggest higher sensitivity than culture in pyogenic spinal infection, specificity is lower, and the underlying studies are heterogeneous; mNGS should therefore complement, not replace, conventional microbiology and histopathology [80].

Targeted next-generation sequencing (tNGS) offers a more focused alternative when the remaining diagnostic question is already relatively narrow—for example, a predefined pathogen panel or a specific resistance question. Its advantages are analytical efficiency and reduced interpretive noise, but performance depends on panel design, tissue adequacy, and local expertise, and the available data are largely single-center and not specific to routine first-line evaluation of spinal biopsy specimens [81,82,92]. tNGS should therefore also be viewed as a selective escalation tool rather than as a default component of initial work-up.

Resource availability substantially affects the practical applicability of the molecular diagnostic pathway described above. Xpert MTB/RIF Ultra is available in many high-TB-burden countries through national tuberculosis programs and WHO procurement mechanisms, but access to the assay for spinal specimens specifically—rather than sputum—varies considerably and may require referral to specialist centers. In low-resource settings where Xpert Ultra is unavailable or inaccessible for extrapulmonary specimens, conventional mycobacterial culture and Ziehl-Neelsen staining with fluorescence microscopy remain the diagnostic backbone for suspected TS, and clinical and histopathological findings carry proportionally greater weight in the diagnostic framework. Metagenomic and targeted next-generation sequencing currently require sequencing infrastructure, bioinformatics expertise, and per-sample costs, rendering them inaccessible for routine use in most low- and middle-income settings. Their use is therefore currently confined to specialist centers in high-income settings, and the molecular pathway proposed in Figure 1 should be adapted to local resource availability rather than applied uniformly.

From a practical perspective, the molecular pathway can be simplified. When TS is plausible, the first biopsy should usually include mycobacterial culture, histopathology, and Xpert MTB/RIF Ultra, with follow-on resistance testing when TB is detected, and resistance information is likely to alter early management. When PS remains likely, but cultures are negative, particularly after antibiotics, 16S rRNA PCR may add incremental value. In unresolved, atypical, or culture-discordant cases, mNGS or tNGS may be considered as selective escalation tools rather than routine first-line tests. In all scenarios, molecular results require interpretation alongside conventional microbiology, histopathology, and pre-test probability [7,9,80].

The diagnostic tests and approaches discussed in this section vary substantially in their evidence base, clinical applicability, and resource requirements. To facilitate practical decision-making and to make the evidential weighting transparent, Table 4 summarizes the main tissue-based and molecular diagnostic parameters, their key performance metrics where available, the study basis from which estimates are drawn, and the evidence-strength classification assigned according to the framework described in the Methods. Quantitative estimates should be interpreted as indicative of the order of magnitude of diagnostic gain rather than as precise calibrated values, given that most derive from single-center retrospective cohorts or heterogeneous study populations without external validation.

## 4. Discussion

The present narrative review synthesizes non-imaging evidence for the differential diagnosis of TS and PS across five clinical domains. Several of its principal conclusions converge with—and extend—existing guidelines and systematic reviews, while others highlight genuine uncertainties that current guidelines do not fully address.

The recommendation to obtain blood cultures prior to antimicrobial therapy and to pursue image-guided biopsy in stable patients is consistent with the 2015 IDSA guidelines on native vertebral osteomyelitis and the 2022 SPILF clinical practice guidelines, both of which emphasize early microbiological diagnosis and deferral of empirical antibiotics where clinically safe [7,25]. Our synthesis reinforces this recommendation while extending it to emphasize parallel tissue allocation for bacterial and mycobacterial culture, histopathology, and molecular testing in a single biopsy session—an approach supported by recent biopsy-yield meta-analyses [70] but not always reflected in older guideline language that predates the widespread availability of rapid molecular platforms.

The role of Xpert MTB/RIF Ultra in extrapulmonary and spinal tuberculosis is endorsed by the WHO 2024 rapid diagnostics module [78], which recommends Xpert MTB/RIF or Ultra for extrapulmonary TB detection and rifampicin-resistance screening. Our synthesis is consistent with this guidance and adds the operationally important point that the assay should be requested at the time of first biopsy rather than reserved for salvage testing—a point not explicitly stated in current guideline language but supported by the prospective data of Waters et al. [67] and consistent with the paucibacillary nature of spinal tuberculosis, which makes repeat sampling both logistically difficult and clinically delayed.

Regarding mNGS, the recent meta-analysis by Li et al. [80] reported pooled sensitivity superior to conventional culture for pyogenic spinal infection, with lower specificity. Our synthesis is consistent with this finding and contextualizes it within a clinical framework: mNGS adds the greatest value in culture-negative, antibiotic-exposed, or diagnostically unresolved cases, rather than as a first-line replacement for conventional microbiology. This position is broadly consistent with the current European Society of Radiology Essentials guidance on radiomics [67] and with recent consensus statements on clinical metagenomics, which similarly recommend NGS as a selective escalation tool.

Several areas of divergence from existing guidance are also worth noting. Current IDSA and SPILF guidelines do not provide specific guidance on the use of composite haematological markers such as NLR in the TS-versus-PS differential, reflecting the limited and retrospective evidence base at the time of their publication. Our synthesis suggests that NLR may offer incremental discriminatory value when interpreted alongside conventional inflammatory markers, but emphasises that the evidence base consists predominantly of single-centre retrospective cohorts without external validation—a limitation that the guidelines’ silence on this topic implicitly reflects. Similarly, the role of IGRA in spinal TB diagnosis is acknowledged in TB-specific guidance but not integrated into spondylodiscitis-specific guidelines; our synthesis positions IGRA as an adjunctive tool that shifts pre-test probability in low-incidence settings rather than a stand-alone diagnostic test.

Taken together, the present review adds value relative to existing comprehensive reviews [1,7,9] primarily in three areas: (1) integration of 2024–2026 molecular diagnostics data, including the Waters et al. Xpert Ultra prospective study and the Li et al. mNGS meta-analysis, within a clinically actionable framework; (2) explicit evidence-strength characterization of each diagnostic parameter using a pre-specified three-dimensional appraisal framework; and (3) a structured synthesis specifically oriented toward the culture-negative or biopsy-delayed diagnostic scenario, which is underrepresented in existing guidelines and review literature despite being one of the most challenging and consequential clinical situations in spinal infection management.

## 5. Limitations of the Review

This review has several limitations that should be considered when interpreting its findings. The search was limited to PubMed/MEDLINE and Scopus; Embase, Web of Science, and Cochrane were not searched due to a lack of institutional access, which may have led to incomplete retrieval of European clinical series and grey literature. Formal dual independent screening was not performed; title and abstract screening was conducted by the lead author with co-author verification on a 20% random sample, introducing a potential source of selection bias. Formal risk-of-bias scoring using standardized instruments—the Quality Assessment of Diagnostic Accuracy Studies 2 (QUADAS-2) tool and the Risk Of Bias In Non-randomized Studies of Interventions (ROBINS-I) tool—was not performed; the three-dimensional appraisal framework applied throughout the Results is a pragmatic alternative that does not fully substitute for structured quality assessment. No Grading of Recommendations Assessment, Development and Evaluation (GRADE)-style evidence grading was applied; the consistent/limited/insufficient convention used in this review is a simplified alternative intended to make evidence strength transparent to readers rather than a formal certainty-of-evidence classification.

Much of the comparative literature is retrospective, enriched for microbiologically confirmed cases from tertiary referral centers, and drawn from a limited number of geographic regions; these features may exaggerate the contrast between TS and PS phenotypes encountered in community-level or resource-limited practice. Pediatric data are limited in quantity and quality, and no pediatric-specific diagnostic algorithm is proposed. The molecular diagnostic pathway presented in Figure 1 has not been prospectively validated and represents an evidence-informed expert synthesis rather than a tested clinical tool. Biomarker thresholds reported in this review—particularly for NLR—derive from single-center retrospective cohorts without external validation and should not be applied uncritically to different populations. The synthesis is also weighted towards adult practice in high-income settings; the diagnostic performance of the assays and strategies discussed may differ in high-TB-burden low-resource settings where reference standards, infrastructure, and pre-test probabilities are substantially different. These constraints reinforce the need to interpret any single diagnostic clue or assay within the broader clinical context.

Furthermore, MRI and CT remain indispensable for confirming an infectious spinal process, identifying epidural or paraspinal extension, and directing biopsy to the most informative target [9,26]. Even highly suggestive imaging, however, cannot replace tissue when management depends on distinguishing TS from PS. Because the present manuscript is the clinicobiological and tissue-based component of a two-part narrative review, detailed MRI/CT/positron emission tomography(PET)-CT differentiation is addressed separately in Part II.

## 6. Conclusions

Differentiating TS from PS rarely depends on a single clinical, laboratory, or imaging feature. In practice, diagnosis emerges from the convergence of epidemiological risk, tempo of illness, inflammatory profile, and, wherever possible, tissue-based confirmation. Features that raise suspicion of TS include chronicity, constitutional symptoms, deformity, and a less intense acute-phase response; features that shift probability towards PS include healthcare exposure, bacteremia, and more marked neutrophilic inflammation. These signals remain probabilistic rather than diagnostic. In stable patients, the most efficient strategy is early blood cultures followed by image-guided biopsy with prospective allocation of tissue for routine microbiology, mycobacterial studies, histopathology, and selected molecular assays. When TS is plausible, Xpert MTB/RIF Ultra has the clearest first-line molecular role; broader sequencing approaches are better reserved for selected unresolved cases. Taken together, this non-imaging synthesis provides the practical framework for Part I of the review, while imaging discrimination is considered separately in Part II.

## Figures and Tables

**Table 2:** High-yield clinical and laboratory clues favoring tuberculous versus pyogenic spondylodiscitis.

Domain	Findings that Favor TS	Findings that Favor PS	Diagnostic Pitfall/Clinical Caveat
**Tempo of illness**	Insidious course over weeks to months	Acute or subacute course over days to a few weeks	Culture-negative PS can also present indolently
**Systemic features**	Weight loss, night sweats, low-grade fever or no fever, psoas abscess, concomitant pulmonary or extra-spinal TB	High fever, rigors, sepsis, obvious extra-spinal pyogenic focus	Absence of fever does not exclude PS
**Neurology/deformity**	Neurological deficit and kyphotic deformity are more frequent because of delayed presentation and collapse	Neurological deficit occurs, but deformity is less typical at first presentation	Urgent decompression decisions remain imaging and examination-driven
**Inflammatory markers**	CRP, ESR, WBC may be normal or only moderately elevated; lower NLR more typical	Higher CRP, WBC, neutrophil count, fibrinogen, and often higher procalcitonin	No single threshold is definitive
**Microbiology**	Blood cultures usually negative; biopsy often paucibacillary; Xpert Ultra particularly useful	Blood cultures more often positive; tissue cultures often guide targeted antibiotics	Prior antibiotics reduce yield in both
**Histopathology**	Granulomatous inflammation, caseous necrosis, giant cells, tubercles	Dense neutrophilic suppuration and purulent necrosis	Atypical suppurative TB exists and can mimic PS

Data synthesized from comparative cohort studies, histopathological series, and reviews cited in the main text. TS (tuberculous spondylodiscitis); PS (pyogenic spondylodiscitis); TB (tuberculosis); CRP (C-reactive protein); ESR (erythrocyte sedimentation rate); NLR (neutrophil-to-lymphocyte ratio); WBC (white blood cell count).

**Table 3:** Tissue-based and molecular tests relevant to the differential diagnosis of tuberculous and pyogenic spondylodiscitis.

Title	Usual Specimen	Typical Turnaround	Best Clinical Role	Main Limitation
**Blood cultures**	Peripheral blood before antibiotics	24–120 h	May establish PS without spinal sampling; should be routine in all cases	Low yield for TS; prior antibiotics reduces positivity
**Conventional tissue culture**	Disc, endplate, vertebral body, paraspinal or epidural collection	2–7 days for routine bacteria; weeks for mycobacterial culture	Core microbiological diagnosis in PS; remains essential in TS (definitive isolation and phenotypic susceptibility testing)	Percutaneous culture yield is often modest; mycobacterial culture is slow and insensitive in paucibacillary disease
**Histopathology**	Biopsy core and curetted tissue	24–72 h	Most useful in culture-negative or partially treated disease; distinguishes granulomatous from pyogenic patterns	Non-caseating or suppurative TB may be non-specific
**AFB stain**	Tissue or pus	Same day	Rapid support for TS when positive	Low sensitivity in paucibacillary spinal TB
**Xpert MTB/RIF Ultra**	Tissue or pus from the first biopsy	Approximately 2 h once run	Fast confirmation of TS and early rifampicin-resistance signal; clearest first-line molecular adjunct when TS is clinically plausible	Negative result does not exclude TS completely
**Line probe assay/follow-on TB molecular DST**	Tissue extract or culture isolate, depending on the platform	Hours to days	Selective follow-on resistance characterization after TB detection	Availability varies; requires adequate nucleic acid; spinal/extrapulmonary data remain limited
**Broad-range 16S**	Biopsy tissue or aspirate	1–3 days	Useful when pyogenic infection is suspected but cultures are negative or prior antibiotics were given	Contamination risk; limited susceptibility information
**Metagenomic NGS**	Biopsy tissue, aspirate, occasionally plasma microbial cell-free DNA	24–48 h	Selective escalation test for culture-negative, polymicrobial, atypical, or unresolved infection	Cost, laboratory availability, host DNA background, false positives, interpretive complexity
**Targeted NGS**	Tissue or isolate with predefined pathogen/resistance panel	24–72 h	Selective escalation tool when a predefined pathogen or resistance question remains unresolved	Restricted to targeted panels; performance depends on panel design and local expertise

Data synthesized from reviews, cohort studies, systematic reviews, and meta-analyses cited in the main text. Turnaround times and local availability are laboratory-dependent. PS (pyogenic spondylodiscitis); TS (tuberculous spondylodiscitis); AFB (acid-fast bacilli); TB (tuberculosis); DNA (deoxyribonucleic acid); DST (drug susceptibility testing); MTB (Mycobacterium tuberculosis); NGS (next-generation sequencing); RIF (rifampicin).

**Table 4:** Summary of diagnostic parameters, performance metrics, and evidence strength for differentiating tuberculous from pyogenic spondylodiscitis.

Parameter	Evidence Strength	Key Performance Data	Study Basis	Clinical Caveat
**ESR elevated**	Consistent	Higher in PS vs. TS across multiple cohorts; no validated cut-off	Multiple multicenter comparative cohorts	Not specific; elevated in both; magnitude helps, threshold does not
**CRP elevated**	Consistent	Higher in PS; [63] (propensity-matched): CRP higher in PS after covariate adjustment	Prospective propensity-matched + retrospective cohorts	Culture-negative PS may overlap with TS profile
**WBC/neutrophil count**	Consistent	Higher in PS; leukocytosis more frequent in PS [19]	Multiple comparative cohorts	Leukopenia does not exclude PS
**NLR < 6.742 for TS**	Limited	Sensitivity 78.3%, specificity 83.6% ([40] no 95% CI reported in primary paper)	Single-center retrospective	No external validation; threshold likely population-dependent
**PLR**	Insufficient	Similar directional trend to NLR; lower discriminatory performance	Single-center retrospective	Not recommended as stand-alone discriminator
**Procalcitonin**	Limited	Higher in PS; overlap substantial; no validated diagnostic threshold	Single-center series	Use to increase confidence in PS when elevated; does not exclude TS when normal
**IGRA (QuantiFERON)**	Limited	Sensitivity 82.6%, specificity 77.8% [66]	Single-center prospective	Cannot distinguish latent from active TB; lower specificity in high-prevalence populations
**Biopsy yield (CT-guided)**	Consistent	Approximately 1/3 of cases microbiologically confirmed (systematic review [70])	Systematic review and meta-analysis	Prior antibiotics reduce yield; antibiotic-free interval of ≥4 days improves yield where feasible
**Histopathology (granuloma)**	Consistent	Caseating granuloma supports TS in ~80% of confirmed cases (n = 181) [72]	Pathology series (single-center)	Minority of confirmed TS lacks granulomas; granulomas also in brucellosis and fungal infection
**Xpert MTB/RIF Ultra**	Consistent	Sensitivity 100% (open biopsy, 95% CI 69–100%), 89% (CT-guided, 95% CI 65–99%) vs. culture; [67]	Prospective single-center	Single-centre data; negative result does not exclude TS; sensitivity lower in paucibacillary disease
**16S rRNA PCR**	Limited	Incremental yield over culture in culture-negative PS; variable across studies	Retrospective cohort studies	Contamination risk; no susceptibility data; results require clinical correlation
**mNGS**	Limited	Higher pooled sensitivity than culture in pyogenic infection; lower specificity [80]	Meta-analysis (heterogeneous studies)	Cost, availability, interpretive complexity; false positives; not first-line
**tNGS**	Insufficient	No spinal-specific validated performance data available	Single-center mixed osteoarticular series	Selective escalation tool only; panel-dependent performance

Evidence strength defined as: Consistent = findings replicated across ≥ 3 independent cohorts with compatible designs; Limited = 1–2 studies or studies with important methodological limitations; Insufficient = no eligible data or data too heterogeneous to support a directional conclusion. Performance data presented as point estimates with 95% CIs, where reported in primary publications. All estimates should be considered indicative rather than definitive. PS (pyogenic spondylodiscitis); TS (tuberculous spondylodiscitis); TB (tuberculosis); CI (confidence interval); CRP (C-reactive protein); CT (computed tomography); ESR (erythrocyte sedimentation rate); WBC (white blood cell); IGRA (interferon-gamma release assay); mNGS (metagenomic next-generation sequencing); NLR (neutrophil-to-lymphocyte ratio); PLR (platelet-to-lymphocyte ratio); RIF (rifampicin); 16S rRNA PCR (16S ribosomal RNA gene polymerase chain reaction); tNGS (targeted next-generation sequencing).

## Data Availability

No new data were generated or analyzed during the preparation of this manuscript. The review synthesizes evidence from previously published studies, all of which are cited in the reference list. Raw data supporting the individual cited studies are available from the respective original publications and their corresponding authors.

## Supplementary Materials

The following supporting information can be downloaded at: https://www.mdpi.com/article/10.3390/diagnostics16142243/s1, Supplementary file S1: Full electronic search strategies used in this narrative review, including the complete Boolean search strings for PubMed/MEDLINE and Scopus with MeSH controlled vocab-ulary terms and free-text operators across three concept blocks; a reference table of all MeSH headings applied; the list of five high-citation narrative reviews hand-searched for additional eligi-ble records; and the pre-specified eligibility criteria and definitions of confirmed and probable spondylodiscitis applied during record screening.

## Abbreviations

The following abbreviations are used in this manuscript:

16S rRNA PCR	16S polymerase chain reaction, ribosomal RNA gene
AFB	Acid-fast bacilli
CD4	Cluster of differentiation 4 (T-lymphocyte surface marker)
CI	Confidence interval
CRP	C-reactive protein
CT	Computed tomography
DNA	Deoxyribonucleic acid
DST	Drug susceptibility testing
ESR	Erythrocyte sedimentation rate
GRADE	Grading of Recommendations Assessment, Development and Evaluation
HIV	Human immunodeficiency virus
IDSA	Infectious Diseases Society of America
IGRA	Interferon-gamma release assay
MDR	Multidrug-resistant
mNGS	Metagenomic next-generation sequencing
MRI	Magnetic resonance imaging
NGS	Next-generation sequencing
NLR	Neutrophil-to-lymphocyte ratio
PCR	Polymerase chain reaction
PLR	Platelet-to-lymphocyte ratio
PRISMA	Preferred Reporting Items for Systematic Reviews and Meta-Analyses
PS	Pyogenic spondylodiscitis
QUADAS-2	Quality Assessment of Diagnostic Accuracy Studies 2
ROBINS-I	Risk Of Bias In Non-randomized Studies of Interventions
SPILF	Société de Pathologie Infectieuse de Langue Française (French Infectious Diseases Society)
TB	Tuberculosis
tNGS	Targeted next-generation sequencing
TS	Tuberculous spondylodiscitis
WBC	White blood cell count
Xpert Ultra	Xpert MTB/RIF Ultra(Cepheid; rapid nucleic acid amplification test for Mycobacterium Tuberculosis and rifampicin resistance)
WHO	World Health Organization
