# Supplementary material for: Differentiating Tuberculous and Pyogenic Spondylodiscitis: Part I—Epidemiology, Clinical Features, Laboratory Markers, and Tissue-Based Diagnosis
Source: Diagnostics (Basel). 2026 Jul 17;16(14):2243. doi: 10.3390/diagnostics16142243 (PMC13409248; doi:10.3390/diagnostics16142243)
Supplement: Supplementary file 1 [file diagnostics-16-02243-s001.zip › diagnostics-4379549-supplementary.pdf]

Supplementary File S1

Full Electronic Search Strategies — PubMed/MEDLINE and Scopus

Differentiating Tuberculous and Pyogenic Spondylodiscitis: Part I. Epidemiology, Clinical Features, Laboratory Markers and Tissue-Based Diagnosis — A Narrative Review

Submitted to Diagnostics (MDPI) | Date of search: 31 March 2026

Important Note on Review Design and PRISMA Compliance

This supplementary file provides the full electronic search strategies used in this narrative review for transparency and reproducibility purposes. As stated in the Methods section of the manuscript, the literature search was conducted iteratively and by topic to support the manuscript's predefined diagnostic domains. Formal de-duplicated title and abstract screening of all records retrieved by broad database searches was not performed. Accordingly, this article is not presented as PRISMA-compliant, and no PRISMA flow diagram is provided. The search strings below represent the structured queries run in each database and are provided to allow readers to independently verify the scope of retrieval and to facilitate replication or updating of the search by future reviewers.

S1. Overview of Search Strategy

The literature search was conducted in two databases — PubMed/MEDLINE as the primary source and Scopus as a secondary supplementary source — using structured Boolean search strings that combined Medical Subject Headings (MeSH) controlled vocabulary with free-text terms tagged to the title and abstract fields ([tiab]). MeSH terms improve retrieval sensitivity by capturing all synonyms used for the same concept, regardless of author terminology; free-text terms capture recent articles not yet fully indexed with MeSH headings. Using both in parallel is standard practice for maximizing recall in biomedical searches.

The search was structured around three concept blocks combined with Boolean AND operators:

Block A — Condition: terms describing the disease entities under review (spondylodiscitis, vertebral osteomyelitis, spinal tuberculosis, and synonyms)

Block B — Pathogen or aetiology: terms identifying the two principal pathogens (Mycobacterium tuberculosis, pyogenic bacteria) and relevant microbiological descriptors

Block C — Diagnostic domain: terms covering the five non-imaging diagnostic domains addressed in this review: biopsy, laboratory markers, histopathology, molecular diagnostics, and immunological tests

A separate pediatric sub-search was run by appending age-group terms to the combined Block A AND Block B string and is provided in Section 3.

S2. PubMed/MEDLINE — Main Search String

Database: PubMed/MEDLINE (US National Library of Medicine)

Interface: https://pubmed.ncbi.nlm.nih.gov

Date range: Database inception to 31 March 2026

Date search was run: 31 March 2026

Filters applied at search stage: None (no language filter, no date limit, no study design filter; all filters applied during manual screening)

Full Search String — copy and paste directly into the PubMed search box

```
(
 ("Discitis"[MeSH] OR "Tuberculosis, Spinal"[MeSH]
  OR "Osteomyelitis"[MeSH]
  OR "spondylodiscitis"[tiab]
  OR "vertebral osteomyelitis"[tiab]
  OR "spinal tuberculosis"[tiab]
  OR "pyogenic spondylodiscitis"[tiab]
  OR "tuberculous spondylodiscitis"[tiab]
  OR "spinal infection"[tiab]
  OR "discitis"[tiab]
  OR "Pott disease"[tiab]
  OR "Pott's disease"[tiab]
 )

AND

 ("Mycobacterium tuberculosis"[MeSH]
  OR "Staphylococcus aureus"[MeSH]
  OR "Kingella"[MeSH]
  OR "pyogenic"[tiab]
  OR "granulomatous"[tiab]
  OR "Mycobacterium tuberculosis"[tiab]
  OR "Staphylococcus"[tiab]
  OR "Kingella kingae"[tiab]
  OR "mycobacterial"[tiab]
 )

AND

 ("Biopsy"[MeSH]
  OR "Molecular Diagnostic Techniques"[MeSH]
  OR "Interferon-gamma Release Tests"[MeSH]
```

OR "C-Reactive Protein"[MeSH]

OR "Blood Sedimentation"[MeSH]

OR "Leukocyte Count"[MeSH]

OR "Procalcitonin"[MeSH]

OR "Histological Techniques"[MeSH]

OR "Nucleic Acid Amplification Techniques"[MeSH]

OR "High-Throughput Nucleotide Sequencing"[MeSH]

OR "Metagenomics"[MeSH]

OR "biopsy"[tiab]

OR "histopathology"[tiab]

OR "culture"[tiab]

OR "Xpert Ultra"[tiab]

OR "Xpert MTB"[tiab]

OR "16S PCR"[tiab]

OR "16S rRNA"[tiab]

OR "metagenomic sequencing"[tiab]

OR "metagenomic next-generation sequencing"[tiab]

OR "mNGS"[tiab]

OR "targeted next-generation sequencing"[tiab]

OR "tNGS"[tiab]

OR "next-generation sequencing"[tiab]

OR "interferon-gamma release assay"[tiab]

OR "IGRA"[tiab]

OR "QuantiFERON"[tiab]

OR "C-reactive protein"[tiab]

OR "CRP"[tiab]

OR "erythrocyte sedimentation rate"[tiab]

OR "ESR"[tiab]

OR "procalcitonin"[tiab]

OR "PCT"[tiab]

OR "neutrophil-to-lymphocyte"[tiab]

OR "NLR"[tiab]

OR "neutrophil lymphocyte ratio"[tiab]

OR "acid-fast"[tiab]

OR "AFB"[tiab]

OR "line probe assay"[tiab]

OR "drug susceptibility"[tiab]

OR "microbiological diagnosis"[tiab]

```
  OR "percutaneous biopsy"[tiab]
  OR "CT-guided biopsy"[tiab]
 )
)
```

Note: [MeSH] searches the controlled MeSH vocabulary and automatically includes all narrower terms (exploded). [tiab] searches title and abstract free text. Boolean operators (AND, OR, NOT) must be in uppercase in PubMed. Parentheses are used to group terms within each block. Copy and paste the entire string, including all outer parentheses.

S3. PubMed/MEDLINE — Pediatric Sub-Search

Run separately to supplement retrieval for the pediatric subsections of §3.1 and §3.3.

```
(
 ("Discitis"[MeSH] OR "Tuberculosis, Spinal"[MeSH]
  OR "Osteomyelitis"[MeSH]
  OR "spondylodiscitis"[tiab]
  OR "spinal tuberculosis"[tiab]
  OR "vertebral osteomyelitis"[tiab]
 )

AND

 ("Mycobacterium tuberculosis"[MeSH]
  OR "pyogenic"[tiab]
  OR "tuberculous"[tiab]
 )

AND

 ("Child"[MeSH] OR "Adolescent"[MeSH]
  OR "Infant"[MeSH] OR "Pediatrics"[MeSH]
  OR "pediatric"[tiab] OR "paediatric"[tiab]
  OR "children"[tiab] OR "child"[tiab]
  OR "adolescent"[tiab] OR "infant"[tiab]
 )
)
```

S4. Scopus — Secondary Search String

Database: Scopus (Elsevier)

Interface: https://www.scopus.com

Date range: Database inception to 31 March 2026

Date search was run: 31 March 2026

Role: Secondary search to supplement PubMed/MEDLINE retrieval and to improve coverage of records not indexed in MEDLINE.

Result: No additional eligible records were identified through Scopus beyond those already retrieved through PubMed/MEDLINE and reference list hand-searching. The Scopus search, therefore, did not yield any additional included records.

```
TITLE-ABS-KEY(
 ( "spondylodiscitis" OR "vertebral osteomyelitis"
   OR "spinal tuberculosis" OR "discitis"
   OR "Pott disease" OR "spinal infection"
   OR "pyogenic spondylodiscitis"
   OR "tuberculous spondylodiscitis" )

 AND

 ( "Mycobacterium tuberculosis" OR "pyogenic"
   OR "granulomatous" OR "Staphylococcus"
   OR "Kingella" OR "mycobacterial" )

 AND

 ( "biopsy" OR "histopathology" OR "culture"
   OR "Xpert Ultra" OR "Xpert MTB" OR "16S PCR"
   OR "16S rRNA" OR "metagenomic sequencing"
   OR "mNGS" OR "next-generation sequencing"
   OR "tNGS" OR "interferon-gamma release assay"
   OR "IGRA" OR "QuantiFERON"
   OR "C-reactive protein" OR "CRP" OR "ESR"
   OR "procalcitonin" OR "neutrophil-to-lymphocyte"
   OR "NLR" OR "acid-fast" OR "AFB"
   OR "drug susceptibility" OR "molecular diagnosis"
   OR "percutaneous biopsy" OR "CT-guided biopsy" )
```

)

Note: Scopus uses TITLE-ABS-KEY() to search title, abstract, and author keywords simultaneously. Boolean operators in Scopus are case-insensitive. Phrase searching uses double quotation marks. Unlike PubMed, Scopus does not use MeSH; all retrieval relies on free-text terms.

S5. MeSH Terms Applied — Reference Table

The following MeSH headings were used with [MeSH] tags in the PubMed search. Unless otherwise noted, each heading is searched in exploded form, meaning PubMed automatically includes all narrower (child) terms in the MeSH hierarchy.

MeSH Heading	Notes on scope and coverage
Discitis	Primary disease heading: includes spondylodiscitis and intervertebral disc infections
Tuberculosis, Spinal	Specific heading for Pott disease and tuberculous spondylitis; narrower than Tuberculosis, Osteoarticular
Osteomyelitis	Broad heading: captures vertebral osteomyelitis when combined with spinal free-text terms in Block A
Mycobacterium tuberculosis	Organism-level heading: includes all M. tuberculosis complex entries
Staphylococcus aureus	Primary pyogenic pathogen in native vertebral osteomyelitis
Kingella	Covers Kingella kingae; important in pediatric spondylodiscitis
Biopsy	Includes CT-guided biopsy, percutaneous needle biopsy, and core needle biopsy subheadings
Molecular Diagnostic Techniques	Broad heading covering PCR, sequencing, and other nucleic acid methods
Interferon-gamma Release Tests	Covers QuantiFERON-TB Gold and T-SPOT.TB assays
C-Reactive Protein	Acute-phase protein; key laboratory marker
Blood Sedimentation	Erythrocyte sedimentation rate (ESR)
Leukocyte Count	White blood cell count (WBC)
Procalcitonin	Sepsis and bacterial infection marker
Histological Techniques	Broad heading for tissue processing and staining methods including acid-fast staining
Nucleic Acid Amplification Techniques	Covers Xpert MTB/RIF Ultra, conventional PCR, LAMP, and related amplification platforms
High-Throughput Nucleotide Sequencing	Covers next-generation sequencing (NGS), whole-genome sequencing, and metagenomic sequencing
Metagenomics	Specific heading for metagenomic approaches, including mNGS

MeSH Heading	Notes on scope and coverage
Child	Pediatric sub-search; covers ages 6-12 years per MeSH definition
Adolescent	Pediatric sub-search; covers ages 13-18 years
Infant	Pediatric sub-search; covers ages 0-23 months
Pediatrics	Pediatric sub-search; broad specialty heading

S6. Hand-Searching — Sources

Reference lists of all comparative TS-versus-PS studies and biopsy-yield cohorts identified through database searches were systematically hand-searched. The following five high-citation narrative reviews published after 2015 were additionally screened in full for eligible references not captured by the database search:

	Full citation of the hand-searched reviews
1	Crombé A, Fadli D, Clinca R, et al. Imaging of spondylodiscitis: a comprehensive updated review — multimodality imaging findings, differential diagnosis, and specific microorganisms detection. Microorganisms. 2024;12(5):893. doi:10.3390/microorganisms12050893
2	Zou X, Li X, He K, Song Q, Yin R. Current knowledge of vertebral osteomyelitis: a review. Eur J Clin Microbiol Infect Dis. 2025;44(2):213-231. doi:10.1007/s10096-024-04983-9
3	Yu D, Kang Y, Lu W, Chen B. Progress in diagnosis and treatment of primary spondylodiscitis: a systematic literature review. EFORT Open Rev. 2025;10:815-828. doi:10.1530/EOR-2025-0041
4	Lacasse M, Derolez S, Bonnet E, et al. 2022 SPILF clinical practice guidelines for the diagnosis and treatment of disco-vertebral infection in adults. Infect Dis Now. 2023;53(3):104647. doi:10.1016/j.idnow.2023.01.007
5	Hirunpat P, Panyaping T, Wongpipathpong W, Hirunpat S. Imaging clues for the diagnosis of various pathogenic causes of infectious spondylitis. Skeletal Radiol. 2025;54:2411-2424. doi:10.1007/s00256-025-04943-0

S7. Eligibility Criteria Applied During Screening

Inclusion criteria

- Original studies reporting clinical, laboratory, histopathological, or molecular diagnostic data in patients with confirmed or probable spondylodiscitis, vertebral osteomyelitis, or spinal tuberculosis
- Studies directly comparing TS and PS patients on at least one diagnostic parameter
- Biopsy-yield or culture-yield studies in vertebral infection, regardless of aetiological breakdown
- Pathology series describing histological findings in spinal infection
- Studies reporting sensitivity, specificity, predictive values, or likelihood ratios for laboratory markers, rapid molecular tests (Xpert Ultra, 16S rRNA PCR, mNGS, tNGS), or immunological tests (IGRA) in spinal or musculoskeletal infection
- Paediatric series (patients aged <18 years) reporting diagnostic data in spinal infection
- Systematic reviews and meta-analyses within the defined scope (used for contextualisation of estimates; primary studies not re-extracted)
- Published in peer-reviewed journals or as national/international clinical guidelines

Exclusion criteria

- Case reports and series of fewer than ten patients, unless reporting diagnostic findings not available in larger studies (retained exceptions are identified as such in the manuscript text)
- Studies restricted to spinal epidural abscess without vertebral body or disc involvement
- Studies exclusively addressing post-operative or implant-associated infection where the TS-versus-PS differential was not applicable
- Imaging-only studies (addressed in the companion manuscript, Part II)
- Non-English records without an available English abstract
- Conference abstracts without accessible full-text data

Definitions of confirmed and probable spondylodiscitis

Confirmed spondylodiscitis: Microbiologically established disease by positive culture of a causative organism or by a validated molecular test (Xpert MTB/RIF Ultra or 16S rRNA gene PCR) from spinal or paraspinal tissue or blood.

Probable spondylodiscitis: Clinical and radiological diagnosis meeting published guideline criteria (IDSA 2015 or SPILF 2022) in the absence of microbiological confirmation. Where the reference standard materially affects the interpretation of reported results, this is explicitly noted in the Results section of the manuscript.
